# Development of Concrete Mixture for Spun-Cast Full-Scale Precast Concrete Pipes Incorporating Bundled Steel and Polypropylene Fibers

**DOI:** 10.3390/ma16020512

**Published:** 2023-01-05

**Authors:** Adeel Faisal, Safeer Abbas, Syed Minhaj Saleem Kazmi, Muhammad Junaid Munir

**Affiliations:** 1 Civil Engineering Department, University of Engineering and Technology, Lahore 54890, Pakistan; 2 School of Engineering, RMIT University, Melbourne, VIC 3001, Australia

**Keywords:** spun-cast method, bundled fiber, three-edge bearing test, crack control

## Abstract

Spin casting is the oldest method of manufacturing precast concrete pipes among all existing methods. While improved concrete mixtures incorporating fibers for other methods of concrete pipe manufacturing, such as the vibration method and roller compaction method, have been developed, no such concrete mixture has yet been developed for spun-cast concrete pipes. This study was designed to explore the possibility of incorporating locally manufactured steel fibers and commercially available polypropylene fibers to develop an improved concrete mixture for use in the manufacturing of full-scale spun-cast concrete pipes. The used steel fibers were of two types, i.e., straight and bundled steel fibers, manufactured by cutting locally available long straight and bundled steel wires, respectively. Various dosages of steel fibers (i.e., 20, 30, 40, and 50 kg/m^3^) and polypropylene fibers (i.e., 5, 10, 15, and 20 kg/m^3^) were used in mono and hybrid (steel and polypropylene) forms. The properties in the fresh state and mechanical properties of the test mixtures were investigated. Full-scale spun-cast concrete pipes having a 450 mm internal diameter were manufactured and tested using the three-edge bearing test. The compressive strength of the mixtures was largely insensitive to the dosage of the fibers. The splitting tensile strength of all fiber-reinforced concrete mixtures was higher than that of the reference mixture without fibers, with a 24% increase recorded for the concrete mixture incorporating 50 kg/m^3^ of bundled steel fibers relative to the reference mixture with no fibers. The flexural performance of the fiber-reinforced concrete mixtures was superior to that of the reference mixture without fibers in terms of flexural strength, toughness, residual strength, and crack control, with up to 28% higher flexural strength relative to the reference mixture without fibers. The three-edge bearing tests on full-scale spun-cast pipes incorporating steel fibers showed that the use of fibers is a promising alternative to the traditional steel cage in spun-cast concrete pipes.

## 1. Introduction

Water is a basic necessity of life, and access to safe water is recognized as a basic human right [1]. Most consumed water becomes wastewater and requires safe disposal [2]. The demand for water supply and consequently the amount of wastewater generated are rising due to growth in population, rise in demand per capita, and expansion in economic activities [3,4]. It is estimated that about 85 billion m^3^ of wastewater is generated annually in North America alone [5]. The worldwide annual generation of wastewater was estimated to be about 380 billion m^3^ in 2020 and is expected to have increased by 24% in 2030 and by 51% in 2050 [6]. Wastewater, if not properly disposed of, poses threat to the environment, as it contains pathogens, which cause many infectious diseases such as diarrhea, typhoid, hepatitis, and cholera [7]. The safe disposal of wastewater involves collection, conveyance, and treatment of the wastewater before releasing it into water bodies [8]. Concrete pipes are important components of wastewater collection and conveyance systems.

Concrete pipes are manufactured by various methods. The most adopted methods are vibration, roller compaction, and the spinning or centrifugal method [9]. These methods differ mainly in the mechanism of consolidating the concrete and the type of concrete used. In vibration method, a vertical pipe mold with an annular space is fed with dry-mix concrete. The concrete is consolidated through intense vibrations and hydraulic pressing from the top. The vertical casting position and dry-mix nature of the concrete allow the pipe to be demolded immediately after casting, as it can support its self-weight [10]. In the roller compaction method, a mold with an internal diameter equal to the required external diameter of the pipe is used. Dry-mix concrete is dropped into the mold. The dry-mix concrete lands on the top of a computer-controlled rotating roller head, equipped with a set of small rotors. The centrifugal action of the small rotors spreads the concrete towards the wall of the mold. The rotating roller head mechanically compacts the concrete against the wall of the mold to form the internal surface of the pipe. The rotating roller head gradually ascends to the top to make the required length of the pipe. The pipe is demolded immediately, and the mold is reused for another pipe [11]. In the centrifugal method, a pipe mold, fitted with end rings, is supported on two rollers. A variable-speed electrical motor is used to rotate the rollers. The rotating rollers rotate the pipe mold. Concrete with an initial water/cementitious materials ratio ranging between 0.40 and 0.50 (depending on whether a water-reducing admixture is used) is fed uniformly into the rotating mold throughout the length, either manually or by a concrete feeder [12]. The concrete mixture must be wet enough to allow the concrete to stick to the walls of the rotating mold. The required wall thickness is controlled by the end rings. When enough concrete has been poured to form the required wall thickness, the speed of rotation is increased. The centrifugal action of rotation pushes the concrete against the wall of the mold, resulting in the extraction of water due to its lower specific gravity than the remaining concrete constituents. Thus, the final water/cementitious materials ratio is decreased. However, the laitance formed on the inner surface of the pipe must be cleaned with the help of a brush. It is important that the initial concrete mixture is not too wet, as otherwise the extraction of excess water will take too long, which not only reduces the efficiency of the process but also results in the drainage of cement paste from the surface of the aggregates, leaving behind weak and porous concrete. The durability of concrete is adversely affected by increased porosity [13,14]. At the end of the rotation, the concrete pipe, along with the mold, is lifted and placed in the curing area. The pipe is demolded after 24 h and cured by ponding or showers.

The manufacturing of concrete pipes by the vibration method and roller compaction method is performed using computer-controlled automatic plants. These methods have the advantages of high efficiency, better quality control due to automation, and high concrete strength due to a low water/cementitious materials ratio. These methods have the additional advantage of producing concrete pipe with uniform concrete strength throughout the wall thickness. Due to these advantages, these methods are popular in North America and Europe. Manufacturing by the centrifugal method is performed either manually or using an automated plant. Due to the high initial investment and technical expertise required for an automated plant, spun-cast pipes are still manufactured manually in developing countries, including Pakistan. It is known that the centrifugal method of manufacturing concrete pipes results in the segregation of concrete ingredients, with larger particles tending to move towards the outer side and the smaller particles towards the inner side [15]. This sets up a gradient in the strength of the concrete over the thickness of the pipe wall such that the strength is higher on the outer side relative to the inner side of the wall [16].

**Figure 1:**
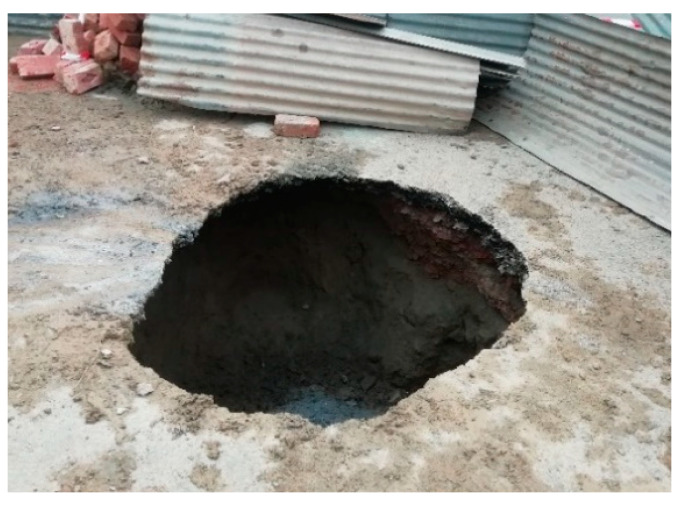
Road collapse due to an underlying pipe failure.

The tensile strength of plain concrete is low, and hence concrete pipes are vulnerable to cracking [17]. As concrete pipes are buried structures, the cracks often go undetected [18]. The cracks pave the way for the corrosion of conventional steel bars by allowing chlorides to reach the surfaces of the bars [19,20,21]. The cracking of a concrete pipe can potentially lead to the final failure of the pipe and associated problems such as road accidents and inconvenience to the public (Figure 1).

Structural fibers such as steel fibers and polypropylene fibers are known to enhance the tensile strength, toughness, and resistance of concrete against cracking [22,23,24,25,26]. Due to the ongoing improvement in the properties of synthetic fibers by the polymer industry, their application in concrete is increasing. For instance, Wang et al. [27] explored the impact of polyoxymethylene fiber on the mechanical performance of seawater–sea-sand concrete at various ages. The incorporation of the polyoxymethylene fibers into the seawater–sea-sand concrete resulted in the improvement of the compressive, splitting tensile, and flexural strength of the concrete, especially at the age of 28 days. Xue et al. [28] suggested that the seawater–sea-sand concrete can be utilized to conserve river sand and fresh water by using polyoxymethylene fibers for improved resistance to early age cracking and enhanced mechanical properties. Fang et al. [29] studied the effects of steel fibers having various geometries and shapes on the mechanical performance of ultra-high-performance concrete (UHPC). It was found that hook-ended steel fibers resulted in better improvement in the tensile performance compared with straight steel fibers. Khan et al. [30] found that aligning the steel fibers in the desired direction using magnetic field while the concrete is still in a fresh state resulted in the significant enhancement of the cracking and ultimate loads of concrete. Abed et al. [31] reported improvement in the flexure and shear performance of self-compacting concrete incorporating steel and polypropylene fibers with increasing dosage of the fibers. The workability reduced with increasing fiber content, although it was still in the permissible range. Wolfel et al. [32] concluded from their study on various types of polypropylene fibers that the efficiency of the fibers could be enhanced by use of higher strength polypropylene fibers with increased surface roughness. Wang et al. [33] studied the effect of various types of fibers on the crack resistance of concrete and reported improved resistance of the polypropylene fiber-reinforced concrete to cracking induced by restrained shrinkage.

Several researchers have explored the use of fibers in concrete pipes produced by either the vibration method or the roller compaction method [34,35,36,37,38,39,40,41,42,43,44,45,46,47,48]. The use of fibers in concrete pipes manufactured by the vibration method or the roller compaction method is relatively straightforward, considering that the aforementioned studies did not report any segregation of fibers from the concrete matrix. Moreover, as mentioned earlier, these methods utilize dry-cast concrete with a low water/cementitious materials (w/cm) ratio. For instance, Peyvandi et al. [44] and Mohamed et al. [43] used w/cm ratios of 0.32 and 0.38, respectively. On the other hand, the spun-cast technique of manufacturing precast concrete pipes involves concrete with a w/cm ratio between 0.4 and 0.5. Studies on inclusion of fibers in spun-cast concrete products are scant. Raju et al. [49] investigated the impact of the addition of steel fibers on the shear resistance of spun-cast concrete piles. The research concluded that the steel fibers could be used to replace conventional shear reinforcement in spun-cast concrete piles. No comprehensive study could be found by the authors in the published literature on the use of fibers in spun-cast concrete pipes. This study is aimed at comprehensively investigating the mechanical performance of concrete incorporating locally manufactured straight steel fibers, novel bundled steel fibers, commercially available polypropylene fibers, and hybrid fibers. Full-scale spun-cast concrete pipes with inside diameters of 450 mm were also manufactured in a local pipe factory with fibers completely or partially replacing the conventional steel cage reinforcement and subjected to the three-edge bearing test (TEBT) in the laboratory.

## 2. Research Significance

The application of fiber-reinforced concrete in manufacturing of precast concrete pipes has been a subject of research for the last two decades. The potential of using steel fibers or polypropylene fibers to replace conventional steel cages for the production of precast concrete pipes has been recognized in the EN standard BS EN 1916 [50], ASTM C1765 [51], and ASTM C1818 [52]. However, the current standards on the subject are still in the developing stage. Unlike the standards for conventionally reinforced concrete pipes such as ASTM C76 [53] which provide detailed guidance for the amount of steel reinforcement required for each class of pipe, the current standards on fiber-reinforced concrete pipes do not provide any detailed guidance such as the dosage of fibers required for a particular class of pipe. Due to the lack of such guidelines, commercial concrete pipe manufacturers have shown reluctance toward adopting fiber-reinforced concrete for the production of precast concrete pipes. Moreover, the current standards for fiber-reinforced concrete pipes have been developed mostly based on studies conducted on the two types of fibers, i.e., steel fibers and polypropylene fibers in mono form. Very scant literature is available on the application of steel and polypropylene fibers in the hybrid form for manufacturing precast concrete pipes. Furthermore, the biggest limitation of the previous studies lies in the manufacturing technique used for the production of the concrete pipes. All previous studies on fiber-reinforced concrete pipes have been conducted on the concrete pipes produced by either the vibration method or the roller compaction method. The spun-cast method for the manufacturing of precast concrete pipes is very commonly adopted in developing countries due to the easy and economical installation of its plants. However, limited study has been reported so far on the application of fiber-reinforced concrete in spun-cast concrete pipes. The potential of incorporating fibers into spun-cast concrete pipes needs to be explored because spun-cast concrete is known to exhibit segregation of concrete ingredients based on their specific gravities [16]. It is important to study the behavior of fibers in spun-cast concrete pipes, as the fibers could segregate from the concrete matrix due to the difference in their specific gravities. There is a further need to investigate other fiber types which were not investigated in previous studies to allow for the incorporation of locally available fibers into precast concrete pipes. Therefore, this study was planned to investigate the effects of various proportions of fibers on the mechanical properties of concrete to be used in the spun-cast method. The studied fibers were locally available straight steel fibers, novel bundled steel fibers, and polypropylene (PP) fibers. Furthermore, hybrid steel and PP fibers were also investigated.

## 3. Materials

The materials used by a local concrete pipe manufacturer for the manufacturing of conventional spun-cast concrete pipes were employed in the current study. Ordinary Portland cement was used in all concrete mixtures. River sand having a fineness modulus of 2.15 and coarse aggregate having a nominal maximum size of 12.5 mm from a local crusher plant were used. The control concrete mixture was designed according to the guidelines of ACI 211.3R [54] with a target compressive strength of 36 MPa and a desired slump of 0 to 25 mm. The selection of the target compressive strength was based on the minimum compressive strength of 27.6 MPa required by ASTM C76 [53] for conventional concrete pipes. The desired slump was chosen to mimic the workability of concrete used for the local manufacturing of spun-cast concrete pipes. The concrete mixture proportions employed in this study are presented in Table 1. 

**Figure 2:**
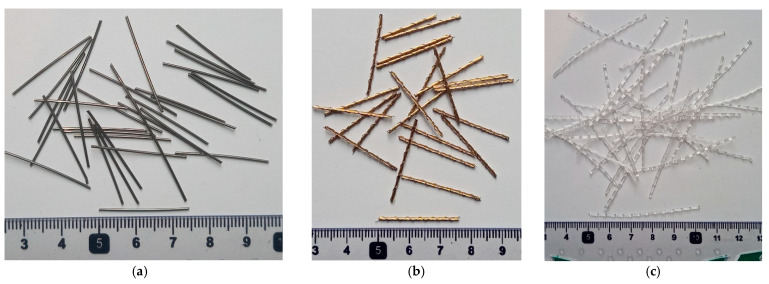
Fibers used: (**a**) steel fibers, (**b**) bundled steel fibers, (**c**) PP fibers.

**Figure 3:**
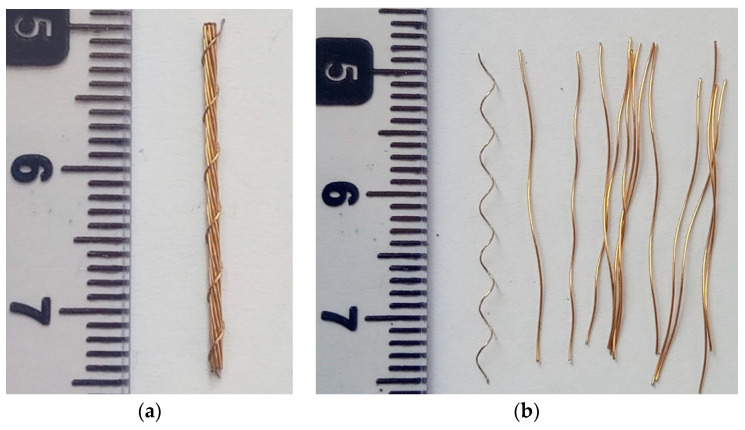
Structure of bundled steel fiber: (**a**) bundled steel fiber, (**b**) unwound steel wires.

Locally manufactured steel fibers of two types and commercially available polypropylene fibers were used (Figure 2). One type of steel fiber was manufactured by cutting locally available straight steel wire having a smooth surface, and the other was manufactured by cutting locally available bundled steel wire into straight lengths of 25 mm. The bundled steel wire (Figure 3) consisted of a strand of 12 interwound thin steel wires wrapped by a steel wire in the form a spiral (Figure 3). Therefore, the bundled steel fiber had a rough surface, suitable for improved bonding with concrete. The fibers employed in this study had the properties given in Table 2. Relatively low dosages of steel fibers and PP fibers were used in this study to avoid the severe reduction in workability of concrete resulting from high dosages of fibers, which could translate into reduced mechanical performance due to the difficulty in achieving the consolidation of concrete [27,28]. The steel fibers were used at dosages of 20, 30, 40, and 50 kg/m^3^ of concrete. The polypropylene fibers were added at dosages of 5, 10, 15, and 20 kg/m^3^.

## 4. Experimental Methodologies

**Figure 4:**
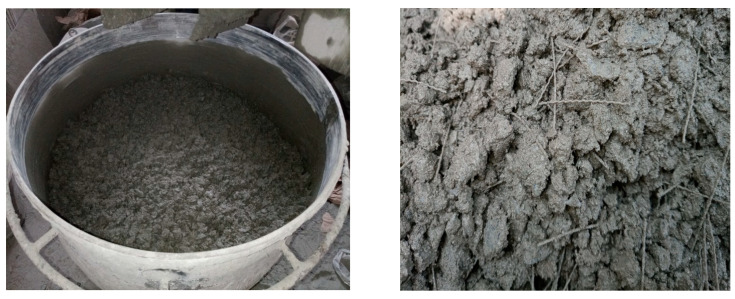
Pan-type mixer used for mixing of concrete.

A pan-type mixer with capacity of 56 L was used for mixing the concrete constituents, (Figure 4). Coarse aggregate, sand, and cement were initially dry-mixed for three minutes. Next, the mixing water was added gradually. The mixing was continued for another three to five minutes. Lastly, fibers were added so as to avoid fiber balling, and the mixing was continued until the fibers were completely dispersed in the concrete.

The slump and the fresh-state density of concrete were measured immediately after the preparation of the mixture in accordance with ASTM C143 [55] and ASTM C138 [56], respectively. For the density of fresh concrete, a 9.3 L capacity cylindrical measure was placed on a vibrating table and filled in two layers. Each layer was given external vibrations for consolidation. Cylindrical specimens with dimensions of 150 × 300 mm and prismatic specimens with dimensions of 100 × 100 × 350 mm were prepared from each mixture. The consolidation of all mixtures was achieved in two layers using a vibrating table. The specimens were demolded after 24 h and cured for 28 days through water ponding to simulate the field curing of small-diameter concrete pipes.

**Figure 5:**
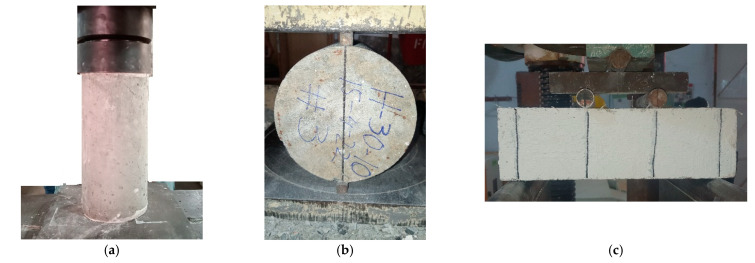
Setups for various tests: (**a**) compressive strength, (**b**) splitting tensile strength, (**c**) flexural strength.

The 150 × 300 mm cylindrical specimens were tested in compression for compressive strength according to ASTM C39 [57]. Plaster of Paris was used to cap the specimens before testing (Figure 5a). Continuous loading at a rate of 0.25 MPa/s was applied to the specimens. Three identical cylinders for compression testing were molded from each mixture to represent the average behavior.

Splitting tensile strength testing was conducted on the 150 × 300 mm cylindrical specimens according to ASTM C496 [58] (Figure 5b). Each specimen was loaded at a rate of 1 MPa/s. Three identical specimens were tested for each mixture.

The flexural performance of the concrete mixtures was assessed in accordance with ASTM C1609 [59] on the 100 × 100 × 350 mm prismatic specimens, (Figure 5c). Displacement-controlled loading at a rate of 0.05 mm/min was applied. The crack width was monitored using leaf gages having various thicknesses, similar to the crack-measuring leaf gauge used in the three-edge bearing test on concrete pipes. Table 3 shows the test matrix.

## 5. Results and Discussion

### 5.1. Material Characterization of Concrete Mixtures Incorporating Fibers 

#### 5.1.1. Fresh Properties

Although the workability of fiber-reinforced concrete mixtures is best determined by vibration-based tests such as Vebe time test, the workability of the concrete mixtures tested in this study was evaluated using the slump test due to the simplicity of the test and its widespread use in the industry. The slump and fresh density results of the tested mixtures are given in Table 4. The slump of the concrete slightly decreased with the increasing dosage of the fibers. For instance, the slump of the mixture with 50 kg/m^3^ of straight steel fibers was 5 mm as compared to 20 mm for the plain concrete mixture. However, the concrete mixture incorporating 20 kg/m^3^ of PP fibers showed zero slump. Similarly, the fresh density of the concrete also marginally decreased as the dosage of the fibers increased. For instance, the fresh density of the concrete mixture incorporating 50 kg/m^3^ of straight steel fibers was about 2% lower than that of the plain concrete mixture. This shows that the slight reduction in the slump resulting from the addition of the fibers in the dosages employed in this study did not significantly affect the level of consolidation of most of the fiber-reinforced concrete mixtures. However, the fresh density of the concrete mixture incorporating 20 kg/m^3^ of PP fibers was about 6% less than that of the plain concrete mixture without fibers. This was because the mixture had a zero slump and could not be consolidated sufficiently.

#### 5.1.2. Compressive Strength

**Figure 6:**
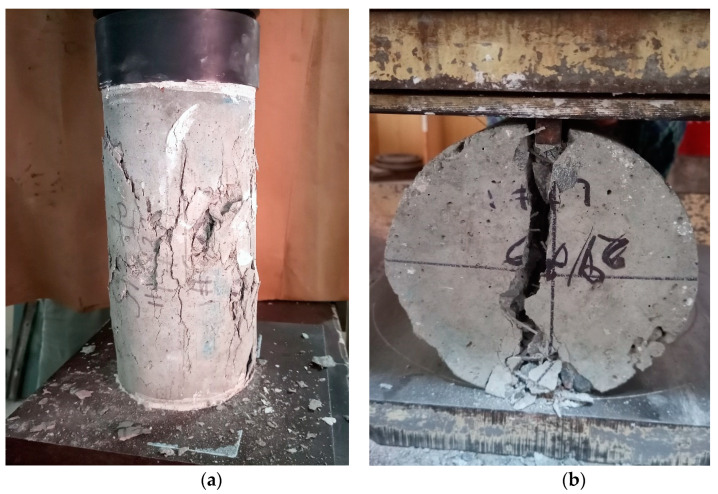
Tested fiber-reinforced cylinders: (**a**) compression test, (**b**) splitting tensile strength test.

The results of the compressive strength tests are given in Table 4. Different trends were observed for the different varieties of fibers employed in this research. In general, the compressive strength of the concrete mixtures incorporating steel fibers (straight or bundled) moderately improved with the addition of the fibers. For instance, the compressive strength of the concrete mixture increased from 34.5 Mpa to 37.1 Mpa when the dosage of the bundled steel fibers was raised from 20 kg/m^3^ to 50 kg/m^3^. A similar trend was observed for the straight steel fibers. The increase in the dosage of the steel fibers increases the likelihood that cracks will be arrested by fibers crossing the cracks, thus leading to somewhat higher compressive strength [60] (Figure 6a). The compressive strength of the mixtures incorporating PP fibers seemed to be insensitive to the dosage of the PP fibers. However, the compressive strength of the mixture having 20 kg/m^3^ of PP fibers was 6% lower as compared to the plain concrete mixture without fibers. This is ascribed to the insufficient consolidation of the mixture due to a loss in workability [61] (slump decreased to zero when the PP fiber content was 20 kg/m^3^).

The dosage of the fibers in the concrete mixtures incorporating hybrid fibers negligibly affected their compressive strength.

#### 5.1.3. Splitting Tensile Strength

The results of the splitting tensile strength tests are listed in Table 4. The splitting tensile strength increased as the dosage of the fibers increased. This was true for all types of fibers employed in this study. For instance, the splitting tensile strength increased from 3.27 MPa to 3.92 MPa (20% increase) when the dosage of the straight steel fibers increased from 20 kg/m^3^ to 50 kg/m^3^. This is ascribed to the fact that raising the fiber dosage increases the number of fibers that can possibly intersect and hold together cracks, thus postponing the splitting of the cylinders [62] (Figure 6b). The bundled steel fibers seemed somewhat more effective as compared to the straight steel fibers. However, the difference in their splitting tensile strengths was only marginal at the same dosage. For instance, the splitting tensile strength of the mixture having the straight steel fibers was 3.73 MPa as compared to 3.78 MPa for the mixture incorporating the bundled steel fibers. This shows that the splitting tensile strength is more sensitive to the dosage of the steel fibers than to the type of the steel fibers used. The splitting tensile strength of the mixtures incorporating PP fibers and those incorporating hybrid fibers (bundled steel fibers and PP fibers) ranged from 1.02 to 1.11 times the splitting tensile strength of the plain concrete mixture without fibers. In comparison, the mixtures incorporating the steel fibers showed splitting tensile strengths ranging from 1.02 to 1.24 times that of the plain concrete mixture without fibers. This shows that the steel fibers were more efficient than the PP fibers and the hybrid fibers in increasing the splitting tensile strength.

#### 5.1.4. Flexural Strength

The results of the flexural tests are presented in Table 5. The peak flexural strength of the fiber-reinforced concrete mixtures was higher than that of the plain concrete mixture, with the increase depending on the dosage of the fibers. This is attributed to the arresting of micro cracks by the fibers, thus delaying the formation of macro cracks [63]. The use of bundled steel fibers resulted in higher flexural strength compared with that of straight steel fibers at the same dosage. For instance, the peak flexural strength increased by 12% and 28% for straight steel fibers and bundled steel fibers, respectively, at the same dosage of 50 kg/m^3^, compared with the plain concrete specimens without fibers. This is possibly because of the relatively roughened surface of the bundled steel fibers formed as the thin individual fibers are interwound to form the bundle, leading to better bonding with the concrete. The PP fibers were found to have a negligible effect on the peak flexural strength with an increase of about 10% at a fiber dosage of 20 kg/m^3^, in agreement with the results of the previous studies [64,65]. 

The concrete mixture incorporating both PP fibers and bundled steel fibers showed better or similar improvement in peak flexural strength compared with the cumulative increase due to the individual fibers. Comparison with the concrete mixtures incorporating either the bundled steel fibers or the PP fibers shows that the enhancement in the peak flexural strength of the hybrid fiber-reinforced concrete mixtures was dependent mainly on the dosage of the bundled steel fibers.

##### Load-Deflection Response

**Figure 7:**
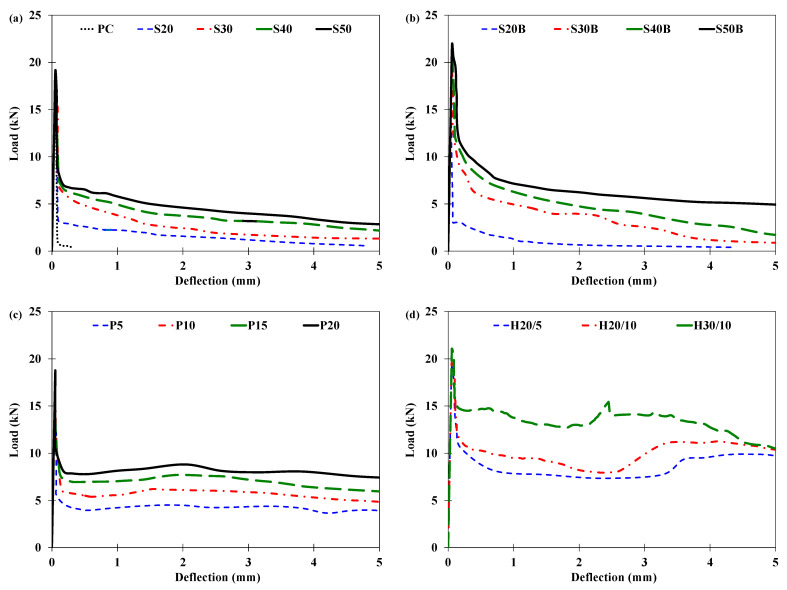
Load-deflection curves in flexure for specimens incorporating (**a**) straight steel fibers, (**b**) bundled steel fibers, (**c**) PP fibers, (**d**) hybrid fibers (bundled steel fibers and PP fibers).

Figure 7 illustrates the load-deflection curves of the prismatic specimens under third-point loading. Initially, as expected, the load increased linearly with the deflection up to the peak load, irrespective of the fiber type and dosage. Following the peak load, all the tested specimens showed a drop in the load with the increasing deflection. The occurrence of the drop in the load coincided with the appearance of a crack in the middle third of the span. The plain concrete specimen abruptly lost its load capacity following the peak load, and the test was terminated. In the case of the fiber-reinforced specimens, the fibers bridged the crack, and the specimens continued to resist the load. However, the specimens incorporating low dosages of fibers (20 kg/m^3^ of the steel fibers or 5 kg/m^3^ of the PP fibers) showed sharp drops in load-carrying capacity after the peak load. This is due to the insufficient number of fibers available to bridge the crack [66]. 

The post-cracking behavior of fiber-reinforced concrete depends on the material, geometry, and dosage of the fibers used [67]. The immediate drop in the load-carrying capacity after the peak load depended on the type of the fibers used. For instance, the load-carrying capacities of specimens incorporating straight steel fibers (50 kg/m^3^), those with bundled steel fibers (50 kg/m^3^), and those with PP fibers (20 kg/m^3^) dropped to 37%, 53%, and 43% of the respective peak loads at a deflection of about 0.17 mm. The specimens incorporating hybrid fibers (30 kg/m^3^ of bundled steel fibers and 10 kg/m^3^ of PP fibers) had a residual strength of about 70% of their peak load at the same deflection of 0.17 mm.

Furthermore, the specimens incorporating straight steel fibers had the steepest rate of loss in residual strength, followed by the specimens incorporating bundled steel fibers. This is possibly because both kinds of steel fibers had a straight geometry with no hooks at the ends, which led to the pullout of the steel fibers from the concrete matrix at low bond stresses [68]. By contrast, the specimens incorporating only PP fibers maintained their residual load-carrying capacities with increasing deflection up to about 5 mm. This was due to the continuously embossed surface of the PP fibers, leading to improved bond performance with the concrete matrix. The specimens incorporating hybrid fibers (PP and bundled steel fibers) initially maintained nearly constant residual strength as the imposed deflection increased, followed by second peaks in the load. The hybrid fiber-reinforced concrete specimens were the only specimens tested in this study which showed second peaks in the load. The occurrence of the second peak was dependent on the dosage of the fibers used in the hybrid fiber system. The increase in the dosage of the fibers resulted in the occurrence of the second peak at lower deflection. All the second peaks in the load were lower than the first peaks, and therefore, the first-peak strength also represented the flexural strength of the specimens.

**Figure 8:**
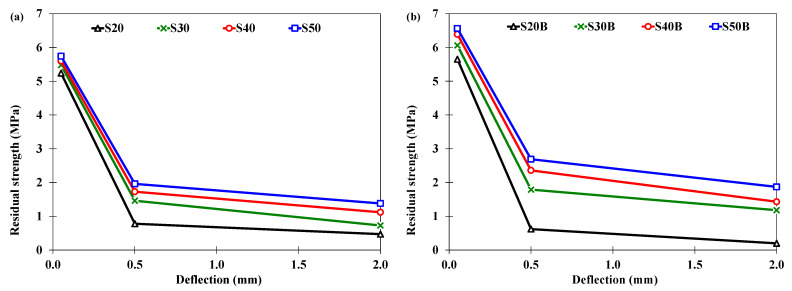

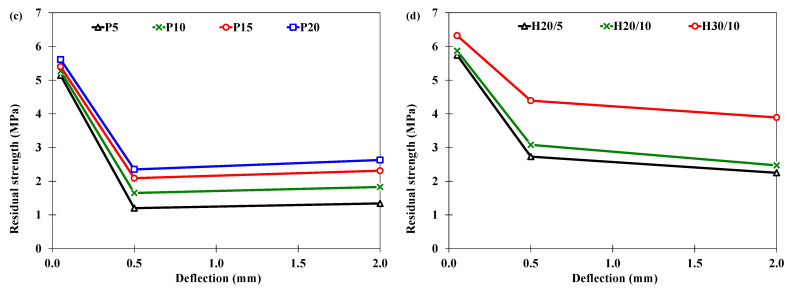
Residual strengths at deflections of L/600 (0.5 mm) and at L/150 (2.0 mm) for specimens incorporating (**a**) straight steel fibers, (**b**) bundled steel fibers, (**c**) PP fibers, (**d**) hybrid fibers (bundled steel fibers and PP fibers).

A comparison of the residual strengths corresponding to deflections of L/600 (0.5 mm) and L/150 (2.0 mm) is given in Figure 8. In general, the residual strength increased with the increase in the dosage of the fibers. However, the type of the fibers used had the most pronounced impact on the residual strength. The bundled steel fibers had higher residual strength as compared to the straight steel fibers. For instance, the residual strengths at L/600 (0.5 mm) and L/150 (2.0 mm) were 1.96 MPa and 1.38 MPa, respectively, for the straight steel fibers, and 2.69 MPa and 1.87 MPa, respectively, for the bundled steel fibers at the same dosage of 50 kg/m^3^. The residual strength of the bundled steel fibers was generally higher at smaller deflection (L/600 = 0.5 mm) as compared to the PP fibers, but lower at larger deflection (L/150 = 0.5 mm). The use of the hybrid fibers (bundled steel fibers and PP fibers) resulted in higher residual strengths at both smaller and larger deflections. For example, the specimens incorporating hybrid fibers (30 kg/m^3^ of bundled steel fibers and 10 kg/m^3^ of PP fibers) resulted in residual strengths of 4.39 MPa and 3.89 MPa at deflections of L/600 (0.5 mm) and L/150 (0.5 mm), respectively.

##### Toughness

Toughness indicates the ability to maintain the load after cracking of the concrete matrix [69]. The toughness was computed by determining the area under the load-deflection curve up to a deflection of L/150 (2.0 mm), following the guidelines of [59]. The toughness of the tested prismatic specimens in flexure is given in Table 5. The toughness increased with the increase in the dosage of the fibers. For instance, the toughness of the bundled steel fibers increased from 3.5 J to 14.1 J when the fiber dosage was increased from 20 kg/m^3^ to 40 kg/m^3^. The straight steel fibers generally had lower toughness as compared to the bundled steel fibers at the same dosage. For instance, the toughness values of the straight steel fibers and bundled steel fibers were 12.0 J and 16.4 J, respectively, at the same dosage of 50 kg/m^3^. This was attributed to the superior post-peak performance of the bundled steel fibers. The PP fibers and the bundled steel fibers showed comparable toughness values at similar levels of dosage of the fibers, except that the bundled steel fibers had much lower toughness (3.5 J) at the first level of dosage used (that is, 20 kg/m^3^). This shows that the dosage of 20 kg/m^3^ of steel fibers is inadequate to improve the post-peak behavior of concrete [43]. The hybrid fibers (bundled steel fibers and PP fibers) outperformed the rest of the fibers used in this study. For instance, the specimen incorporating hybrid fibers (30 kg/m^3^ of bundled steel fibers and 10 kg/m^3^ of PP fibers) had a toughness of 27.6 J, which is the highest among all the specimens tested in this study.

##### Crack and Failure Pattern

**Figure 9:**
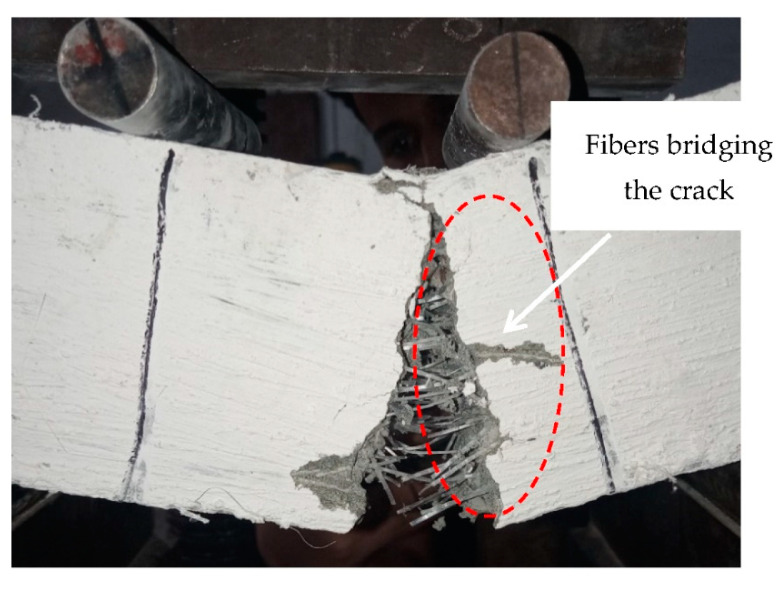
Fibers bridging the crack that appeared in the central third of the specimen tested in flexure.

**Figure 10:**
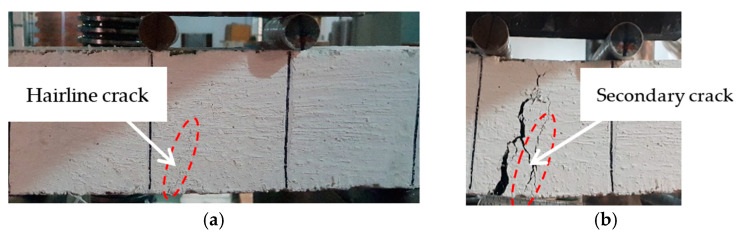
Cracking in the specimen incorporating 20 kg/m^3^ of PP fibers: (**a**) first hairline crack, (**b**) secondary crack.

All the tested specimens showed no cracking during the initial loading phase in which the load increased linearly with the deflection. At the end of the linear elastic phase of the loading, all the tested specimens showed a crack in the central third of the span. As the imposed deflection increased, the crack width increased, and the crack propagated towards the compression face. Nearly all the tested specimens developed a single crack, and multiple cracking was not observed (Figure 9). Only the specimen reinforced with 20 kg/m^3^ of PP fibers showed a secondary crack at a spacing of about 25 mm from the primary crack (Figure 10). The secondary crack appeared when the residual load was about 37% of the peak load. The formation of single cracks in the tested specimens is due to the relatively low fiber dosage employed in this study [70].

**Figure 11:**
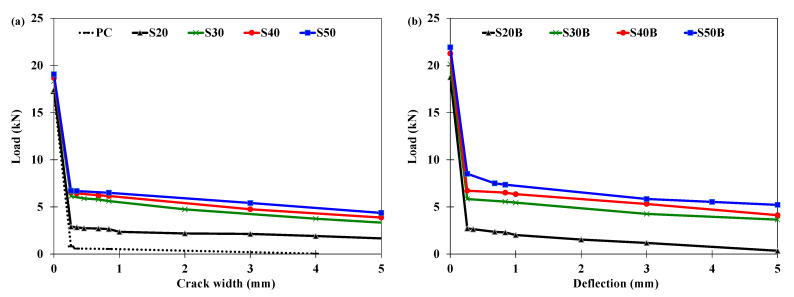

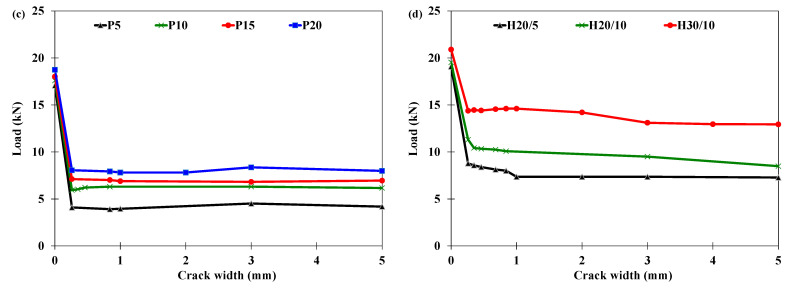
Load vs. crack width recorded at discrete points during the testing for specimen incorporating (**a**) straight steel fibers, (**b**) bundled steel fibers, (**c**) PP fibers, (**d**) hybrid fibers (bundled steel fibers and PP fibers).

Considering the intended application of the concrete mixtures for manufacturing spun-cast full-scale concrete pipes, the crack width was measured at discrete points during loading and plotted against the load (Figure 11). The crack propagation and widening consisted of two phases. In the first phase, unstable crack propagation and widening took place immediately after the initiation of the crack. The second phase was characterized by stable crack propagation and widening [71]. It is worth mentioning that the specimens without fibers and those with low fiber dosages (20 kg/m^3^ of steel fibers or 5 kg/m^3^ of PP fibers) displayed a prominent unstable crack propagation phase, and a loud tick sound was heard when the crack appeared and quickly propagated towards the compression face. The increase in the dosage of the fibers enhanced the ability of the specimen to hold the crack, and thus, mostly stable crack propagation was observed. The first point of the load vs. crack width curves corresponds to the peak load at which the crack is barely visible. The next points on the load vs. crack width curves depict the ability of the specimen to resist crack opening. In general, the load vs. crack width curves confirm the earlier findings on the basis of the load vs. deflection curves. The bundled steel fibers were more efficient in holding a crack as compared to the straight steel fibers. For instance, the loads corresponding to 0.26 mm crack width were 8.51 kN and 6.72 kN for the bundled steel fibers and the straight steel fibers, respectively. The specimens incorporating hybrid fibers (bundled steel fibers and PP fibers) were the most effective in controlling the crack width. The load at 0.26 mm crack width was 14.38 kN for the specimen incorporating hybrid fibers (30 kg/m^3^ of bundled steel fibers and 10 kg/m^3^ of PP fibers).

**Figure 12:**
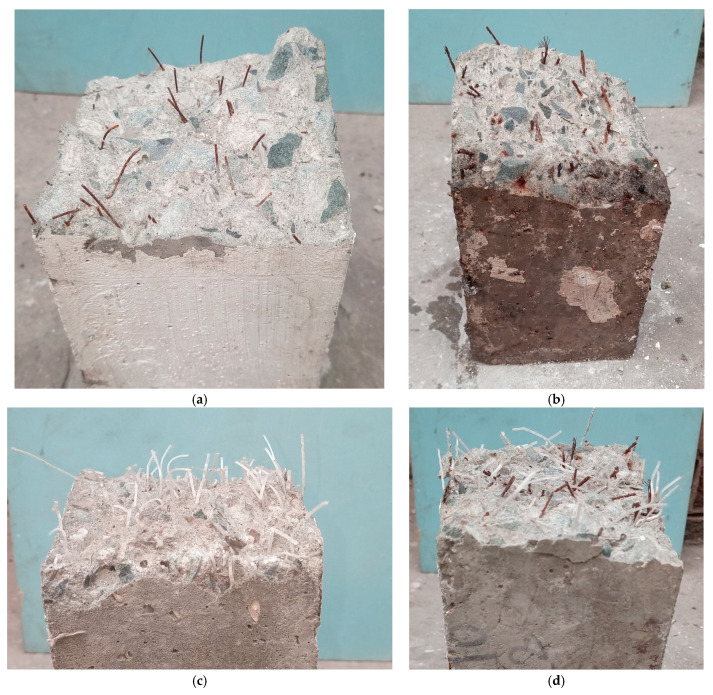
Fracture surfaces of specimens incorporating various fibers: (**a**) straight steel fibers, (**b**) bundled steel fibers, (**c**) PP fibers, (**d**) hybrid fibers.

Furthermore, visual examination of the fracture surfaces of the specimens indicated that both the straight and the bundled steel fibers predominantly failed by fiber pullout, (Figure 12a,b). This is perhaps due to the high strength of the steel fibers and the lack of end anchorage. By contrast, the failure of the PP fibers was primarily due to fiber rupture (Figure 12c). This could be due to the relatively lower strength of the PP fibers and improved bonding with concrete matrix due to their continuously embossed surface.

### 5.2. Manufacturing and Testing of Full-Scale Spun-Cast Concrete Pipes

To study the potential of incorporating the fibers into the manufacturing of full-scale spun-cast concrete pipes, full-scale pipes, having a 450 mm inside diameter and a 63 mm wall thickness, were manufactured at a local facility using the spinning technique. Concrete pipes are traditionally reinforced with a steel cage consisting of circumferential and longitudinal bars. For this study, initially, concrete pipe without any conventional cage was manufactured, incorporating straight steel fibers at a dosage of 20 kg/m^3^. The objective of this initial trial was to observe the behavior of the steel fibers in the spinning process. Accordingly, the concrete ingredients were mixed in a drum-type mixture according to the proportions given in Table 1. After dry-mixing of coarse aggregate, fine aggregate, and cement for two to three minutes, the mixing water was introduced, and wet mixing was performed for another three to five minutes. The straight steel fibers were then gradually added to the mixer so as to avoid fiber balling. The mixing was continued until the steel fibers were dispersed in the concrete mixture. The prepared concrete mixture was unloaded onto the casting yard, from where it was manually poured into the pipe mold with shovels. 

**Figure 13:**
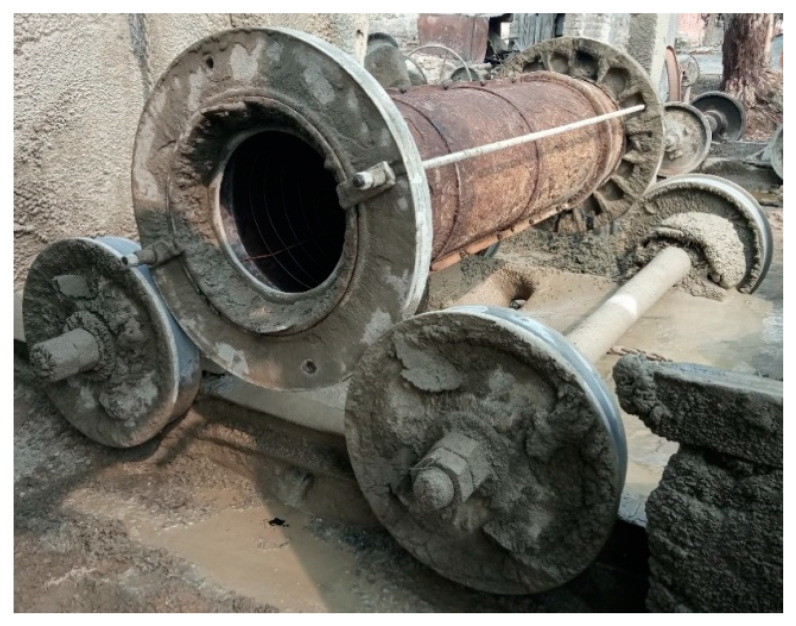
Pipe mold on rotating wheels.

The pipe mold was mounted on two rollers which induced rotating motion to the mold (Figure 13). Initially, the mold was set into rotation at a speed of 160 rpm to 180 rpm. In a conventional concrete pipe, a reinforcing steel cage is inserted into the mold, which helps retain the fresh concrete as it is poured into the spinning mold. The exclusion of the conventional steel cage from the pipe for this study presented a challenge in ensuring the fresh concrete would stick to the wall of the mold. As the fresh concrete was poured into the mold, some of the concrete stuck to the wall of the mold, while some of it fell off. The problem was overcome by reducing the initial spinning speed to 130 rpm to 140 rpm, so as to allow the concrete to stick to the wall of the mold. As the thickness of the pipe wall built up with more concrete poured into the mold, the concrete could bear higher spinning speed without falling off. Therefore, the spinning speed was increased back to 160 rpm to 180 rpm. The pouring of the concrete was stopped when the desired wall thickness was attained. Finally, the spinning speed of the mold was increased to 230 rpm to 250 rpm. It is the centrifugal forces resulting from the spinning process that result in the consolidation of concrete. The water, being the lightest of all the ingredients of concrete, was forced out of the concrete. The water was then drained out with the help of a brush. The expulsion of water from the concrete has the advantage of a reduced water/cementitious material (w/cm) ratio in the final concrete. The spinning was continued until no more water could be extracted from the concrete.

**Figure 14:**
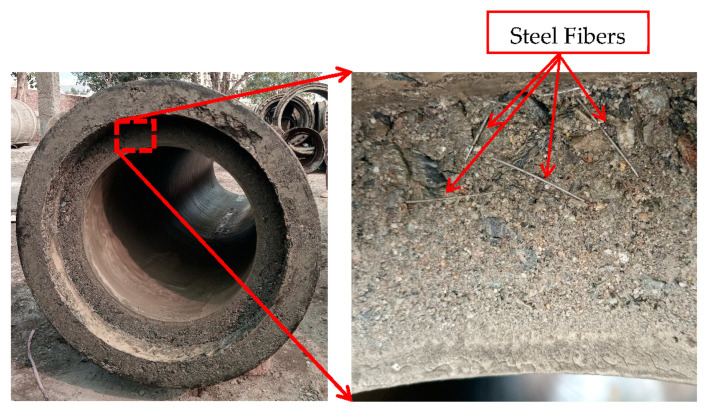
Distribution of steel fibers seen in the freshly cast concrete pipe.

The centrifugal forces presented the biggest challenge to maintaining the uniform dispersion of the steel fibers in the spinning concrete. The visual inspection of the freshly cast concrete pipe revealed that the steel fibers, having much higher specific gravity (7.85), segregated towards the outer side of the wall of the pipe and that no steel fiber could be seen on the inside of the wall of the pipe (Figure 14).

In the second phase, four additional concrete pipes having an inside diameter of 450 mm and a wall thickness of 63 mm were manufactured. Plain concrete pipe without any reinforcement, conventional reinforced concrete pipe, concrete pipe with a conventional steel cage as well as 20 kg/m^3^ of the straight steel fibers, and concrete pipe with a conventional steel cage as well as 40 kg/m^3^ of the straight steel fibers were cast. The conventional steel reinforcement comprised six longitudinal bars of 4 mm diameter and circumferential rebar of 4 mm diameter spaced at 82 mm. 

**Figure 15:**
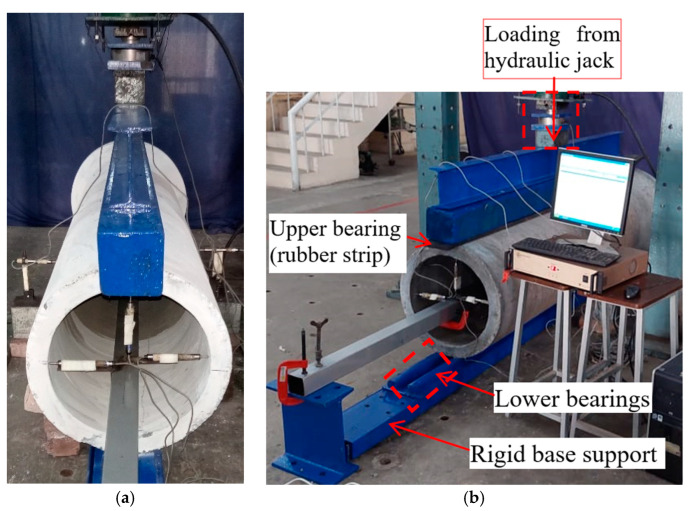
Setup of three-edge bearing test: (**a**) side view, (**b**) 3D view.

**Figure 16:**
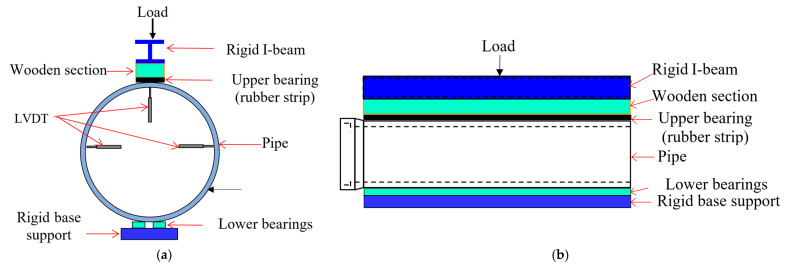
Schematic of three-edge bearing test: (**a**) cross-sectional view, (**b**) longitudinal view.

**Figure 17:**
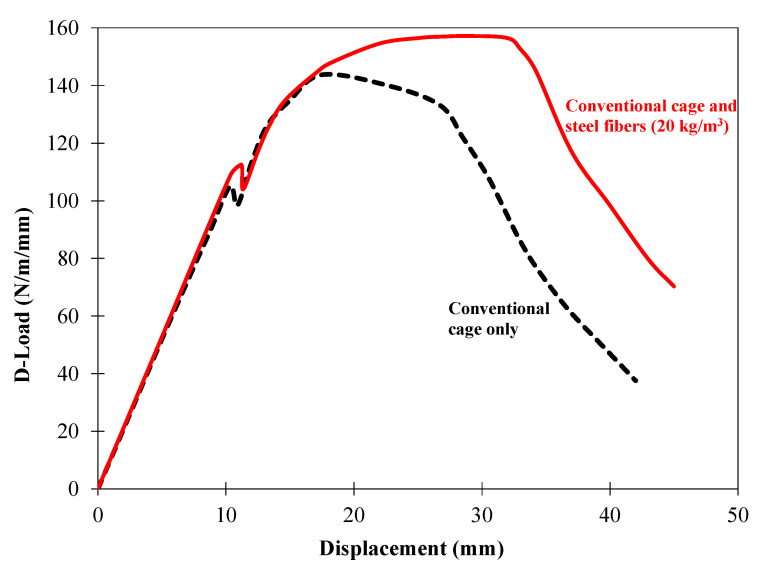
Load-deflection curves of the pipes tested under the three-edge bearing condition.

**Figure 18:**
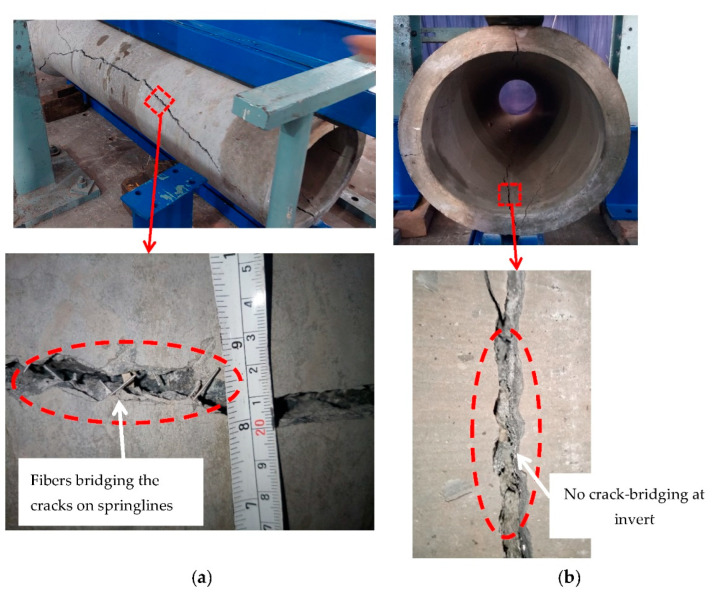
Cracking pattern of the tested concrete pipe incorporating steel fibers: (**a**) crack along springlines, (**b**) crack at invert.

**Figure 19:**
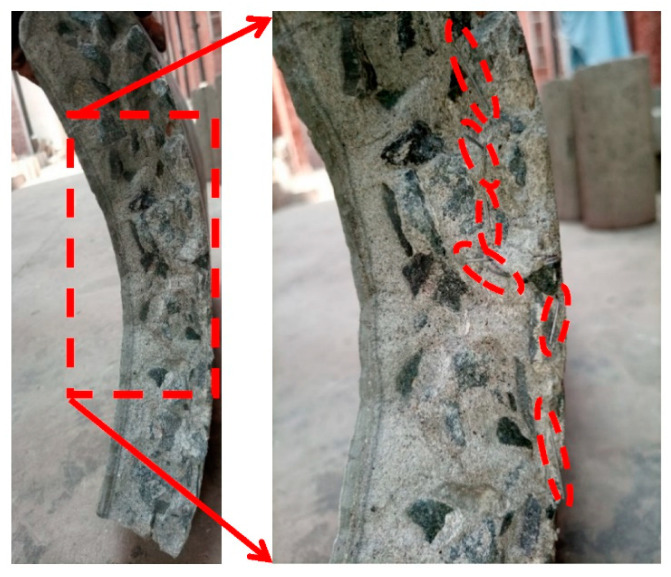
Fracture surface of the tested concrete pipe incorporating steel fibers (Location of steel fibers mentioned at outer edge enclosed in red dashed lines).

The pipes were tested in three-edge bearing loading (Figure 15 and Figure 16), following the guidelines of ASTM C497 [72]. The D-loads corresponding to 0.3 mm crack width and the ultimate load recorded for the tested pipes are given in Table 6. The D-load is the load per unit length per unit diameter of the pipe. Figure 17 shows the typical load-deflection curve for the tested pipes. The inclusion of the steel fibers in the concrete pipes improved both the cracking load (0.3 mm crack width) and the ultimate load. This was due to the ability of the steel fibers to bridge a crack formed along the springlines, (Figure 18a). However, no such crack bridging was observed for the cracks long the invert and the crown of the pipe, (Figure 18b). This was because the steel fibers had relocated towards the exterior side of the wall of the pipe during the spinning process. This was further confirmed by visual inspection of the fracture surface of the pipe (Figure 19). It can be seen that steel fibers were present closer to the outer side of the wall.

## 6. Conclusions

This study explored the mechanical properties of various concrete mixtures incorporating steel and PP fibers in mono and hybrid forms at varying dosages with the aim to incorporate the fibers into concrete mixtures for the manufacturing of full-scale spun-cast concrete pipes. The following specific conclusions can be drawn from this study:

- The dosage and type of fibers generally showed little or no effect on the compressive strength of the fiber-reinforced concrete mixtures. However, some improvement in the compressive strength of up to 18% was observed for the concrete mixture incorporating 50 kg/m^3^ of bundled steel fibers, as compared to the plain concrete mixture without fibers.
- The splitting tensile strength and flexural strength of all fiber-reinforced concrete mixtures were consistently higher as compared to the plain concrete mixture without fibers. The concrete mixture incorporating hybrid fibers (30 kg/m^3^ of bundled steel fibers and 10 kg/m^3^ PP fibers) exhibited much better improvement in flexural strength (24% higher), residual flexural strength at small and large deflections (4.39 MPa at L/600 and 3.89 MPa at L/150, as compared to nil for plain concrete mixture), and toughness (23 times higher) in comparison with the plain concrete mixture without fibers.
- The fibers can be incorporated into concrete mixtures for the manufacturing of full-scale spun-cast concrete pipes without significant changes in the manufacturing process. The tests on full-scale pipes showed that the use of fibers as partial or complete replacements for the conventional steel cage is a viable option to enhance the crack resistance of concrete pipes and eliminate or reduce the time-consuming fabrication of conventional steel cages.
- The use of hybrid fibers consisting of high-specific-gravity fibers (such as steel fibers) and low-specific-gravity fibers (such as PP fibers) can be an effective strategy to deal with the tensile stresses on both the inner and outer sides of the wall of a concrete pipe a with thickness too small to accommodate two layers of conventional rebar.

## Figures and Tables

**Table 1:** Concrete mixture design.

Ingredient	Mass/Cement Mass
Cement	1.00
Fine aggregate	1.50
Coarse aggregate	2.92
Water	0.47

**Table 2:** Properties of used fibers.

Fiber Type	Shape	Length (mm)	Diameter (mm)	Aspect Ratio	Tensile Strength (MPa)	Modulus of Elasticity (GPa)
Steel fibers	Solid fiber with smooth surface and straight ends	25	0.68	36	1569	200
Steel fibers	Bundled fiber and straight ends	25	0.79 *	32	1428	200
PP fibers	Embossed surface	55	0.91 *	60	460	9

PP = Polypropylene fiber; * Equivalent diameter.

**Table 3:** Test matrix.

Sr. No.	Mixture Designation	Fiber Dosage (kg/m^3^)		Mixture Type	Remarks
		Steel	PP		
1	PC	0	0	Plain concrete	Control mix
2	S20	20	0	Steel fiber-reinforced concrete	Straight steel fibers
3	S30	30	0		
4	S40	40	0		
5	S50	50	0		
6	S20B	20	0		Bundled steel fibers
7	S30B	30	0		
8	S40B	40	0		
9	S50B	50	0		
10	P5	0	5	PP fiber-reinforced concrete	Polypropylene fibers with embossed surface
11	P10	0	10		
12	P15	0	15		
13	P20	0	20		
14	H20/5	20	5	Hybrid fiber-reinforced concrete	Hybrid of bundled steel fibers and PP fibers
15	H20/10	20	10		
16	H30/10	30	10		

PC = Control concrete mixture without fibers; S20 = Concrete mixture having 20 kg/m^3^ of straight steel fibers; S30B = Concrete mixture having 30 kg/m^3^ of bundled steel fibers; P5 = Concrete mixture having 5 kg/m^3^ of polypropylene fibers; H20/5 = Concrete mixture having hybrid bundled steel fiber (20 kg/m^3^) and PP fibers (5 kg/m^3^).

**Table 4:** Results of various tests.

Sr. No.	Mixture Designation	Slump (mm)	Fresh Density (kg/m^3^)	Compressive Strength (MPa)	Splitting Tensile Strength (Mpa)
1	PC	20	2513	32.6	3.21
2	S20	15	2502	34.5	3.27
3	S30	10	2493	35.2	3.47
4	S40	5	2486	36.5	3.73
5	S50	5	2474	37.1	3.92
6	S20B	15	2498	33.9	3.32
7	S30B	15	2491	34.4	3.49
8	S40B	10	2482	36.8	3.78
9	S50B	10	2477	38.4	3.98
10	P5	10	2498	32.9	3.29
11	P10	5	2488	31.8	3.35
12	P15	5	2476	31.5	3.48
13	P20	0	2412	30.8	3.57
14	H20/5	10	2495	32.4	3.29
15	H20/10	5	2484	33.1	3.37
16	H30/10	5	2473	34.1	3.55

**Table 5:** Results of flexure tests.

Sr. No.	Mixture Designation	First-Peak Strength (f_1_) and Deflection (δ_1_)		Residual Strength (MPa) at Deflection of		Toughness (Joule)
		f_1_ (MPa)	δ_1_ (mm)	L/600	L/150	
1	PC	5.11	0.05	0	0	1.2
2	S20	5.24	0.05	0.78	0.47	5.2
3	S30	5.48	0.05	1.46	0.73	8.6
4	S40	5.6	0.05	1.73	1.12	10.5
5	S50	5.74	0.05	1.96	1.38	12
6	S20B	5.65	0.05	0.62	0.2	3.5
7	S30B	6.06	0.05	1.79	1.18	11.2
8	S40B	6.39	0.06	2.36	1.43	14.1
9	S50B	6.56	0.06	2.69	1.87	16.4
10	P5	5.14	0.05	1.2	1.34	9.1
11	P10	5.27	0.05	1.65	1.83	12.1
12	P15	5.4	0.05	2.09	2.31	14.7
13	P20	5.61	0.05	2.35	2.63	16.6
14	H20/5	5.74	0.05	2.73	2.25	17.4
15	H20/10	5.87	0.05	3.08	2.47	19.9
16	H30/10	6.32	0.06	4.39	3.89	27.6

**Table 6:** Results of three-edge bearing tests.

Pipe	Reinforcement Details	Steel Fibers (kg/m^3^)	D-Load_0.3mm crack_ (N/m/mm)	D-Load _ult_ (N/m/mm)
1	-	-	60	60
2	-	20	72	72
3	Conventional steel rebar	-	105	140
4	Conventional steel rebar	20	114	155
5	Conventional steel rebar	40	130	185

## Data Availability

Not applicable.
